# Importins and Exportins Regulating Allergic Immune Responses

**DOI:** 10.1155/2014/476357

**Published:** 2014-03-09

**Authors:** Ankita Aggarwal, Devendra K. Agrawal

**Affiliations:** Department of Medical Microbiology & Immunology and Center for Clinical & Translational Science, Creighton University School of Medicine, Omaha, NE 68178, USA

## Abstract

Nucleocytoplasmic shuttling of macromolecules is a well-controlled process involving importins and exportins. These karyopherins recognize and bind to receptor-mediated intracellular signals through specific signal sequences that are present on cargo proteins and transport into and out of the nucleus through nuclear pore complexes. Nuclear localization signals (NLS) present on cargo molecules to be imported while nuclear export signals (NES) on the molecules to be exported are recognized by importins and exportins, respectively. The classical NLS are found on many transcription factors and molecules that are involved in the pathogenesis of allergic diseases. In addition, several immune modulators, including corticosteroids and vitamin D, elicit their cellular responses by regulating the expression and activity of importin molecules. In this review article, we provide a comprehensive list of importin and exportin molecules and their specific cargo that shuttled between cytoplasm and the nucleus. We also critically review the role and regulation of specific importin and exportin involved in the transport of activated transcription factors in allergic diseases, the underlying molecular mechanisms, and the potential target sites for developing better therapeutic approaches.

## 1. Introduction

The trafficking of molecules between the cytoplasm and the nucleus in eukaryotes is regulated by nuclear pore complexes (NPCs or nucleoporins), which are cylindrical structures containing about 100 different polypeptides and embedded in the double membrane of the nuclear envelope. The regulation of bidirectional movement of molecules within a cell is critical in the exchange of molecules in and out of the nucleus and precise control of signal transduction processes as well as gene expression, cell cycle progression, and other cellular responses. Generally, the molecules up to 38–50 kDa in size may passively diffuse through the nuclear pore complexes. However, molecules larger than 50 kDa require machinery whereby mobile targeting receptors called karyopherins recognize and bind to receptor-mediated intracellular signals through specific signal sequences that are present on substrate proteins. Depending upon the movement of macromolecules from cytoplasm to nucleus or nucleus to cytoplasm, these karyopherins target the substrates that contain nuclear localization signal (NLS) or nuclear export signals (NES). The classical amino acid sequence in the NLS is PKKKRRV that mediates the nuclear translocation of large molecules from the cytoplasm [1]. The nuclear export signals (NES) typically contain the sequence LQLPPLERLTL, which directs the proteins to leave the nucleus [2].

**Figure 1:**
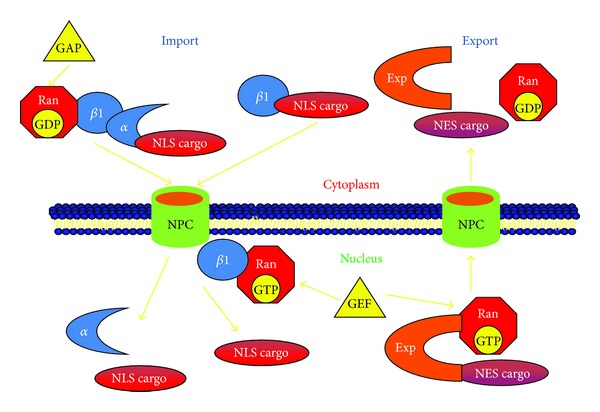
Nucleocytoplasmic transport of macromolecules via importin-exportin pathway. In the cytoplasm, importin-*α* forms a heterotrimeric complex with importin-*β*1, RanGDP, and NLS containing cargo protein. This heterotrimeric complex passes through the NPC into the nucleus and RanGTP binds to importin-*β*1 and disassembles the complex in the nucleus. The binding of exportin to NES within the cargo protein is triggered by RanGTP in the nucleus and is exported back to the cytoplasm through nuclear pore complex (NPC). In the cytoplasm, the dissociation of the complex is mediated through the hydrolysis of RanGTP to RanGDP by GAP (GTPase activating protein) that forces the binding of GDP to Ran. GEF (guanine nucleotide exchange factor) is a nucleoplasmic factor that stimulates exchange of GDP with GTP forming RanGTP in the nucleus.

The first conventional NLS is recognized by an adaptor protein, importin-*α* (also known as karyopherin-*α*), that functions as the NLS receptor. The importin-*α* interacts with importin *β* (also known as karyopherin-*β*), which mediates the process of docking to the nucleoporins. The protein containing NLS is transported through NPC to the nucleus by importin *α*/*β* heterodimeric complex. This translocation of the substrate-receptor complex through the NPC requires energy provided by a low molecular weight Ras-family GTPase, Ran, which is present in the nucleus. After translocation to the nucleus, the importin heterodimeric complex dissociates by the action of Ran-GTP, whereby the importin-*α* and the substrate enter and accumulate in the nucleoplasm while importin-*β* binds to Ran-GTP and accumulates at the NPC [2]. Thus, the import of protein substrates bound to NLS receptors in the cytoplasm does not require Ran-GTP. However, the release of the protein substrate in the nucleus is dependent upon Ran-GTP binding to the complex. Finally, importin *α*/*β* returns back to the cytoplasm to import additional protein molecules into the nucleus (Figure 1).

Nuclear export of proteins occurs through NES in an analogous manner by exportins, which are homologous to importin-*β*. However, this requires the binding of Ran-GTP to protein-NES-exportin complex. After the cytoplasm has been reached, the hydrolysis of RanGTP to RanGDP by Ran GTPase-activating protein occurs, resulting in the dissociation of the complex of exportin and protein. The direction in which protein is carried is generally determined by the gradient of Ran GTPase because the majority of RanGDP and Ran-GTPase-activating proteins are found in the cytoplasm, whereas Ran-GTP and Ran-guanine nucleotide exchange factors (Ran-GEF) are primarily present in the nucleus [3].

Human importins and exportins are classified into four subfamilies. First family is importin *α*P that consists of importin *α*1 [4], second family is importin *α*Q, including importins *α*3 and *α*4 [5], third family is importin *α*S including importins *α*5, *α*6, and *α*7 [5–7], and the fourth family is importin *β* that consists of importins *β*1, *β*2, and *β*3, importins 7, 8, and 12, exportins 1, 5, and 6, and exportin t [8]. However, there are few human importin/exportin molecules that have not been yet categorized into a subfamily. These are importins 4, 9, 11, and 13, transportin 2, and exportins 4 and 7. Different subfamily members share around 50% sequence identity. The similarity in sequence within a subfamily is around 85%. Despite of these subtle differences in the sequence, they possess similar pattern of recognition of NLS on cargo proteins. However the experimental evidence is provided by many studies validating the fact that different importin/exportin molecules possess distinct properties in terms of interacting with the NLS of specific cargo proteins [6, 9, 10]. A comprehensive summary of human importins and exportins along with their alternative names, short names, and the gene symbols given by NCBI (National Center for Biotechnology Information) is provided in Table 1. There also are nonconventional nuclear transport mechanisms that are distinct from the classical concept of importing proteins which are responsible for the import of molecules into the nucleus by directly binding to specific importin *β*1 homologs from importin *β* family.

Karyopherins recognize various cargo molecules and bind to them by interacting with NLS or NES present on cargo molecules. However, the specificity of importins or exportins for each cargo would depend upon the structure and direct interaction of importin-cargo molecules. In Table 2, a summary of cargos that bind directly to importins and exportins is provided.

## 2. Molecules Involved in Allergic Immune Response

**Figure 2:**
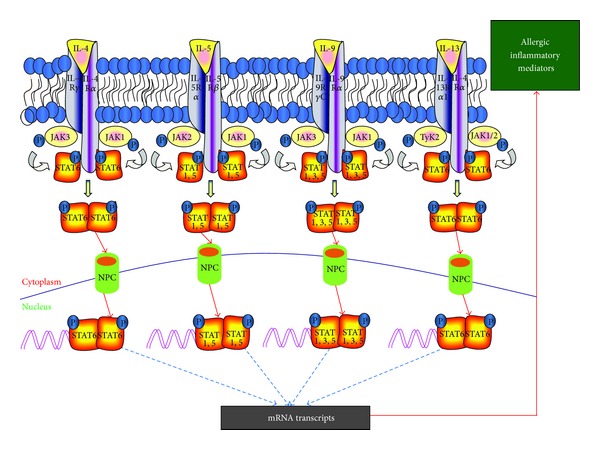
Schematic diagram showing the involvement of transcriptional factors in response to key mediators of allergic inflammation: binding of Th2 cytokines to their specific receptors and the activation of the downstream JAK/STAT pathways specific to each cytokine are shown. JAK: Janus activated kinase; NPC: nuclear pore complex; STAT: signal transducer and activator of transcription; TyK2: tyrosine kinase 2.

Inflammation is a hallmark of allergic diseases, including asthma, allergic rhinitis, and atopic dermatitis. In most of the allergic diseases, inflammation is primarily characterized by the predominance of Th2 lymphocytes and the specific cellular response of Th2 cytokines, IL-4, IL-5, IL-9, and IL-13. IL-4 mediates the class switching of IgM secreted by plasma cells to allergen specific IgE, IL-5 is a major cytokine involved in eosinophilic inflammation, and IL-13 is partly involved in class-switching event and plays a key role in goblet cell hyperplasia and mucus production [11]. IL-9 was initially described as a growth factor for T-lymphocyte and mast cells and is released from many cell types, including Th2 cells, eosinophils, mast cells, and neutrophils. IL-9 can induce multiple effects in the initiation, exacerbation, and maintenance of allergic airway inflammation and airway remodeling (Figure 2).

Patients with allergic respiratory diseases have a biased immune response towards Th2 phenotype, contrasting that of healthy individuals in which the host-defense mechanisms maintain a balance between Th1 and Th2 phenotypes. The differentiation of Th2 cells is regulated by zinc finger transcriptional factor GATA-3, suggesting a key role of GATA-3 in mediating allergic immune response [12]. There is a pivotal role of bronchial smooth muscle cells in the pathogenesis of airway inflammation [13]. The increase in bronchial smooth muscle mass narrows the airway lumen, which further obstructs the airflow. There are a number of inflammatory cytokines secreted by bronchial smooth muscles such as TNF-*α*, IL-6, IL-17, IL-8, and IL-1*β*. Indeed, nearly every structural cell in the lung, including epithelial cells and infiltrated cells, becomes activated during allergic immune response. Thus, airway inflammation, due to the effect of various cytokines and mediators released by the cells in the lung, is a hallmark of allergic diseases.

The transcription, translation, and release of cytokines during allergic response depend upon specific signals, activation of intracellular kinases, and transcription factors present in the cytoplasm (Figure 2). STAT6 is vital to inducing the expression of Th2 cytokines, initiating allergic immune responses in many allergic diseases [14, 15]. The activated NF-*κ*B is one of the transcriptional factors that trigger airway hyperresponsiveness and allergic airway inflammation by rapidly inducing the expression of various genes involved in the pathology of allergic diseases [16]. However along with AP-1, it can further enhance the production of proinflammatory cytokines such as IL-1 and TNF-*α* [17]. Th17 cells are another subset of T helper cells that are differentiated from naïve T cells in the presence of TGF-*β*, and a very potent inflammatory cytokine, IL-6, is credited with inducing pathology in allergic inflammatory diseases [18]. ROR*γ*t is a transcriptional factor responsible for the differentiation of Th17 cells via signal transducer and activator of the transcription 3 (STAT3)-ROR*γ*t pathway. The activation and functionality of transcriptional factors determine the fate of T cell differentiation and the cytokine secretion according to the lineage of the T cell. Th1 cells express Th1 master regulator, T-box transcriptional factor (T-bet), which is induced via STAT4 or STAT1 signaling pathways [19]. Runx3 is another transcriptional factor whose expression is upregulated by Th1 cells. If the conditions favoring the differentiation of Th1 cells are present, then the differentiation of Th2 cells will be inhibited. IL-4 encoding genes are silenced by T-bet and Runx3 [20], suggesting that targeting IL-4 signaling is one of the therapeutic approaches to attenuating allergic airway inflammation. On the other hand, Th2 cell differentiation is accompanied by IL-4 through STAT6, which in turn induces GATA-3 the master regulator for Th2 cell differentiation [21].

The activation of the respective transcriptional factors is a key component to the induction or alleviation of allergic inflammation. The transcriptional factors responsible for inducing or reducing allergic inflammation must enter the nucleus of the target cell and bind to their specific response elements to exhibit their functional role. Small molecules up to ~40 kDa can easily diffuse through the nuclear pore complexes, whereas the larger molecules require help from import and export molecules to get through the nuclear membrane. The import-export machinery responsible for such translocation consists of importins and exportins. In the following section, the role of specific importin or exportin molecules in the transport of specific transcription factors involved in allergic inflammation will be discussed.

## 3. Effect of Importins/Exportins on Key Transcription Factors Involved in Allergic Inflammation Rel or NF-*κ*B

**Figure 3:**
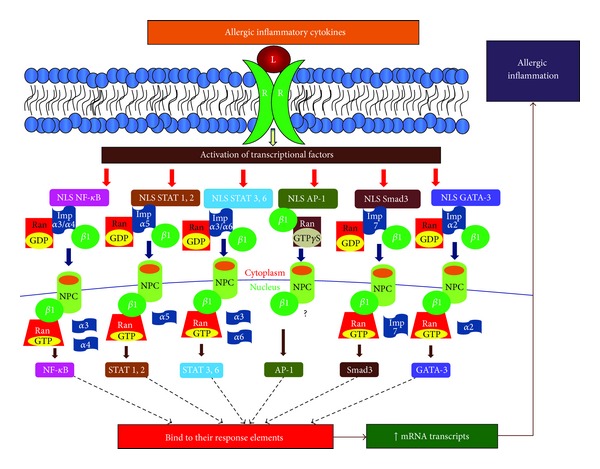
Binding of specific importin molecules to transcription factors: the figure shows the recognition of nuclear localization signal (NLS) by specific importin (Imp) molecules on activated transcriptional factors in the cytoplasm, binding of importin *β*, and transport of the cargo to the nucleus through nuclear pore complex (NPC).

Since importins and exportins play a crucial role in transporting macromolecules into and out of the nucleus, their regulation is critical in cellular responses (Figure 3). Rel/NF-*κ*B p50/p65 is actively involved in cell differentiation, host immune response and in the transcription of many inflammatory cytokines [22]. Rel/NF-*κ*B activation is critical for the induction and stimulation of allergic airway inflammation. Indeed, increased activation of activated Rel/NF-*κ*B has been found in immune cells and structural cells of the lungs in asthmatic subjects [23, 24]. TNF-*α*, IL-17, and IL-1*β* are crucial proinflammatory cytokines that are secreted by many cells in the airway during allergic airway inflammation and activate NF-*κ*B [24]. In the latent state, Rel/NF-*κ*B remains in the inactive state complexed with I*κ*B-IKK complex in the cytosol. IKK complex consists of three distinct subunits: IKK*α* and IKK*β* as catalytic subunits and IKK*γ* (also called as NEMO) as a regulatory subunit to sense scaffold and to integrate the upstream signals to activate catalytic subunits. Once signaled, IKK complex is activated, leading to phosphorylation of IKK*β*. This results in the ubiquitination of I*κ*B*α* followed by degradation by the 26S proteasome. This releases the Rel/NF-*κ*B dimers containing primarily p50–p65 subunits to enter the nucleus and activate target gene expression. Due to the large size of Rel/NF-*κ*B dimers, the translocation event is triggered by importins (Figure 3). NF-*κ*B subunits p50 and RelA contain classical nuclear localization signals that are unmasked after ubiquitination and degradation of I*κ*B*α* prior to their translocation to the nucleus by importin *α*/*β* heterodimer [25].

Fagerlund and colleagues have presented strong evidence in their study to suggest that importins *α*3 and *α*4 are responsible for the translocation of TNF-*α*-stimulated active subunits of NF-*κ*B to the nucleus from the cytoplasm [25] and the subsequent export back into the cytoplasm with the support of exportin, CRM1 (chromosome region maintenance 1) [26, 27]. This exportin has sequence similarity with karyopherin *β*1 and interacts with nucleoporin Nup214 and exports NF-*κ*B to the cytoplasm, thereby inhibiting its accumulation in the nucleus. Thus, importin *α*3 and importin *α*4 together with CRM1 are critical karyopherins involved in the import and export of NF-*κ*B in nucleocytoplasmic translocation during allergic airway inflammation (Figure 3).

Recently, several molecules have been identified which might have a significant effect on CRM1-dependent export of NF-*κ*B and retention of NF-*κ*B in the nucleus. These molecules include prohibitin [11, 27, 28], HSCARG [29], poly(ADP-ribose)polymerase-1 [30], and heat shock protein 72 (Hsp72) [31]. However, the potential presence of additional endogenous mediators as a natural defense mechanism in response to NF-*κ*B activity cannot be ruled out. The underlying mechanisms by which these molecules influence import nucleocytoplasmic shuttling of NF-*κ*B are unclear and warrant further investigation. Nonetheless, there is a potential to develop novel therapeutics in controlling allergic inflammation and the treatment of allergic diseases due to increased activity of NF-*κ*B.

## 4. AP-1

Activator protein-1 (AP-1) is another transcriptional factor that plays a crucial role in the induction of cellular differentiation, gene expression, and apoptosis. Active AP-1 enhances the secretion of proinflammatory cytokines, including TNF-*α* and IL-1. In response to allergic inflammatory mediators, both AP-1 and NF-*κ*B are activated and these transcription factors can synergistically induce the transcription and generation of inflammatory mediators to induce allergic and autoimmune diseases. AP-1 consists primarily of two components, c-jun and c-fos, and the active AP-1 is made up of homodimer or heterodimer of these subunits [32]. The homodimer or heterodimer of activated AP-1 enters into the nucleus primarily through importin *β*1 that recognizes subunits of AP-1 in importin-*α*-independent manner (Figure 3). In fact, Importin *β*1 binds to AP-1 subunits, jun and fos, with higher affinity than that of importin-*α* [33]. Thus, by controlling the binding of importin *β*1, the nuclear translocation of AP-1 may be regulated and could thus be useful in the attenuation of inflammation in allergic diseases.

There are no reports in human cells in regard to the export of AP-1. However, in yeast it has been found that *β*-karyopherin-like nuclear exporter, Crm1p, recognizes and binds to the nuclear export sequence on AP-1 in the presence of RanGTP, and this process is inhibited by oxidation [34].

## 5. GATA-3

GATA-3 is a transcriptional factor that mediates the differentiation and proliferation of Th2 cells [35]. Th2 cytokines, IL-4, IL-5, IL-9, and IL-13, are predominantly regulated by GATA-3 and critically involved in the pathogenesis of allergic airway inflammation. IL-4 and IL-13 are mainly responsible for inducing antigen specific IgE while IL-5 is involved in eosinophilic inflammation. GATA-3 plays a pivotal role in mediating allergic diseases, such as allergic rhinitis, asthma, and atopic dermatitis, and therefore is a key target in developing better therapeutic approaches. Indeed, the knockdown of GATA-3 under both* in vivo* and* in vitro* conditions reduces Th2 cytokines, resulting in inhibited allergic inflammation [36, 37]. GATA-3 contains a classical nuclear import signal that is recognized by importin *α* and thus translocates to the nucleus [37]. The deletion of the region, which is critical for the interaction of the NLS in AP-1 and importin *α*, hinders nuclear translocation of activated AP-1 [38]. The p38 MAPK-mediated serine phosphorylation on GATA-3 is critical for nuclear import of GATA-3 from cytoplasm after its interaction with importin *α* [37]. Upon arrival in the nucleus, GATA-3 binds to its response element in the promoter region of Th2 cytokines, increases their gene expression, thus stimulates IgE class switching by increased production of IL-4 and IL-13, and induces eosinophilic inflammation (Figure 3).

An endogenous inhibitor, MAPK phosphatase-1 (MKP-1), inhibits the phosphorylation and activation of p38 MAPK [37]. The inhibition of p38-MAPK leads to the downregulation of Th2 cytokines [39]. The interaction between importin *α* and NLS on GATA-3 is affected due to the inhibition of p38 MAPK, resulting in the attenuation of nuclear translocation of GATA-3 from cytoplasm (Figure 3). This, in turn, leads to the downregulation of Th2 cytokines and reduced allergic airway inflammation. The results from animal studies also support the fact that the inhibition of the phosphorylation of p38 MAPK causes a reduction in allergic airway inflammation. This is further supported by findings in which the suppressed activity of p38 MAPK reduced eosinophilic inflammation in mice and guinea pigs that were exposed to OVA [40]. Mice sensitized and challenged with OVA were aerosolized with the SB239063, a potent inhibitor of p38 MAPK inhibitor, showed reduced eosinophilic inflammation, and attenuated airway hyperresponsiveness and mucus production, and there was downregulation in IL-4, IL-5, and IL-13 in the bronchoalveolar lavage fluid. These findings suggest that GATA-3 is regulated via its nuclear import gene importin *α*. Thus, potential inhibitors of importin *α* binding to the NLS on GATA-3 could be therapeutically useful in regulating allergic inflammation.

There is no data available at this time on the export of GATA-3 from nucleus to cytoplasm. However, the potential role of CRM-1 cannot be ruled out.

## 6. STAT6, STAT1, and STAT2

Th2 cytokines, IL-4 or IL-13, activate signal transducer and activator of transcription (STAT) 6 by binding to their receptor IL-4R*α* that mediates JAK1 and JAK3, and further activates STAT6 classical pathway. The critical role of STAT6 on IgE class switching, Th2 differentiation, and IL-4 mediated responses is supported by the fact that such effects are drastically impaired in STAT6 knockout mice [41]. STAT6, upon tyrosine phosphorylation, becomes active and translocates to the nucleus by importin *α*-importin *β*1 receptors (Figure 3). STAT6 also possesses the binding sites for importins *α*3 and *α*6 [42]. Little is known about the STAT6 complex crystal structure; however, the data available in the literature suggests that it can be regulated by targeting the importins responsible for its translocation into the nucleus. This suggests an important role of inhibiting or downregulating importins to alleviate allergic diseases.

Both STAT1 and STAT2 are activated in response to type I interferons, IFN-*α*, IFN-*β*, and IFN-*ω*. Both STAT1 and STAT2 have an arginine/lysine-rich nuclear localization signal. The tyrosine-phosphorylation on Tyr-701 and Tyr-69 on STAT1 and STAT2, respectively, results in the dimerization of STAT1-STAT2, and this heterodimer translocates to the nucleus. The NLS on STAT1-STAT2 heterodimer is recognized by and binds to two importin *α*5 molecules that are responsible for their translocation into the nucleus (Figure 3) [43].

## 7. STAT3, ROR*γ*t, and Smad3

IL-6, a proinflammatory cytokine responsible for differentiating Th0 cells into pathogenic Th17 cells, activates STAT3 transcription factor. STAT3 is also responsible for the Th17 cell differentiation. It binds to importin *α*3 and importin *α*6 and is actively transported into the nucleus with the help of these karyopherins [44]. It has also been shown that nuclear import of STAT3 occurs independent of tyrosine phosphorylation. According to another study, it has been shown to have binding sites for importin *α*5 and importin *α*7 as well; however, importin *α*7 has weak interactions with STAT3 [45] (Figure 3).

IL-6 and TGF-*β* contribute to induce the expression of ROR*γ*t (transcriptional factor, required for the initiation and survival of Th17 cell). Other than our knowledge of its involvement in mediating allergic immune diseases, there is no information on its nuclear versus cytoplasmic movement. However, there is no karyopherin-recognizable nuclear localization sequence present on ROR*γ*t [46, 47]. Hence, the translocation of ROR*γ*t from the cytoplasm into the nucleus is carried by S6K2, a nuclear counterpart of S6K1, which is induced by PI3 K-Akt-mTORC1 axis [47]. The S6K2 possess functional classical NLS at its C-terminus. It binds to ROR*γ*t and then carries it into the nucleus from cytoplasm. Since PI3 K-Akt-mTORC1 axis suppresses Gfi1 expression to positively regulate Th17 differentiation and enhances the S6K2 expression responsible for nuclear translocation of ROR*γ*t, the PI3K-Akt-mTORC1 axis-S6K2 is a novel target to limit the differentiation of pathogenic Th17 cell differentiation and could thus be a useful therapeutic approach to control allergic inflammation.

Smad3 is activated in response to TGF-*β*, which is not only a key factor involved in the expression of ROR*γ*t and differentiation of Th17 cells, but also critical in the resolution phase of inflammation by induction of fibrosis at the site allergic reactions. Both importin *β* and importin 7 are utilized in the transport of activated Smad3 from cytoplasm to nucleus [48, 49] (Figure 3).

## 8. Allergic Immune Regulation by Importins and Exportins

Recently, we found that vitamin D decreases airway hyperresponsiveness and allergic airway inflammation via its receptor VDR (vitamin D receptor) that acts to downregulate importin *α*3 in ovalbumin-sensitized and -challenged mice [11]. Although there are several mechanisms proposed in the literature on the beneficial effect of vitamin D in alleviating allergic immune responses, one of the potential targets is importins [50]. Calcitriol, an activated form of vitamin D, stimulates VDR and exerts its beneficial effect by suppressing cell growth, cell proliferation and cell differentiation, and immune regulation. VDR interacts with its response elements in the nucleus and alters the transcription of the subsequent target genes involved in the biological response. We also found that vitamin D reduces allergic responses by increasing the protein expression of prohibitin, a molecule that regulates the activity of importins and its binding to inflammatory transcription factors [51].

Prohibitin (PHB) is a protein that is expressed ubiquitously in cell and mitochondrial membranes and in the nucleus. It is a multifunctional protein that is implicated in various regulatory functions such as proliferation, differentiation, apoptosis, transcription, and protein folding [52]. The expression of PHB decreases in the tissues under inflammatory conditions, including inflammatory bowel disease and airway inflammation, suggesting its potential role as an anti-inflammatory gene [53]. Overexpression and/or restoration of PHB in intestinal epithelial cells downregulated importin *α*3, an importin involved in NF-*κ*B nuclear translocation [27]. Recently, under both* in vitro* and* in vivo* conditions, we found the downregulation of importin *α*3 at both the mRNA and protein levels by prohibitin in response to the activation of calcitriol-activated VDR [11, 34]. In vitamin D-deficient allergic asthmatic mice there was a significant reduction in prohibitin due to an upregulation of importin *α*3 and thus increased translocation of activated NF-*κ*B from the cytoplasm into the nucleus. In vitamin D-sufficient and vitamin D-supplemented mice, the VDR compartments remain intact to keep sufficient amount of prohibitin levels for controlling cellular activity. This regulates importin *α*3-mediated nuclear translocation of NF-*κ*B and thus reduces allergic airway inflammation and airway hyperresponsiveness in response to allergen challenge in allergen-sensitized mice [11]. Thus, in inflammatory conditions, a decrease in expression of either or both VDR and PHB leads to an increase in importin *α*3 expression and its activity, resulting in the exaggeration of allergic inflammatory response.

**Figure 4:**
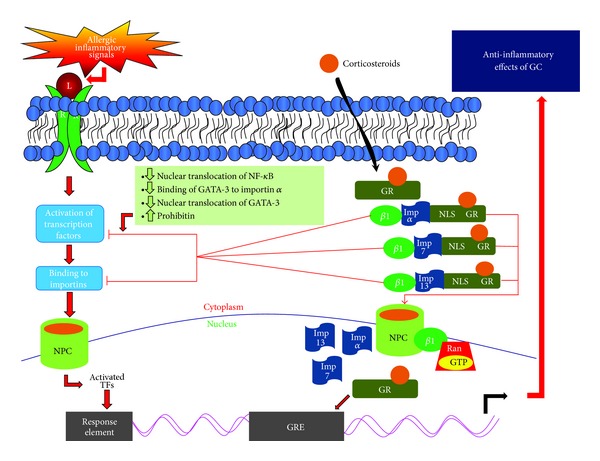
Activation of glucocorticoid receptor (GR) and control of allergic inflammation:corticosteroids are lipophilic in nature and cross the bilipid membrane to bind to their receptors in the cytoplasm. The nuclear localization signal (NLS) on steroid-receptor complex can be recognized by importin *α*, importin 7, or importin 13 to transport the steroid-receptor complex to the nucleus to bind to glucocorticoid-response element (GRE) to induce the transcription of several genes that elicit anti-inflammatory effect. Also, the steroid-receptor complex in the cytoplasm can also induce effects on the nuclear transport of activated transcription factors by either inhibiting the binding of activated transcription factors to importins or increasing the inhibitory molecules, such as prohibitin, in the cytoplasm.

Corticosteroids are potent anti-inflammatory agents and regulate the activity of several transcriptional factors involved in allergic inflammation [54]. Although there could be several potential mechanisms for the underlying anti-inflammatory effects of corticosteroids, one of the major effects is the inhibition of nuclear translocation of activated transcription factors, including GATA-3 and NF-*κ*B (Figure 4). Corticosteroids are highly efficient in suppressing Th2 cytokines in the airways of individuals with allergic asthma. The anti-inflammatory effect by corticosteroids is exerted via binding to cytosolic glucocorticoid receptors (GR). After interacting with their receptors, corticosteroid-GR complex translocates to the nucleus from the cytoplasm where they bind to the promoter region of steroid-sensitive genes containing glucocorticoid response elements (GREs). The activated glucocorticoid receptors suppress inflammatory action exerted by NF-*κ*B via interaction with coactivator molecules. This is achieved by inhibiting either the nuclear translocation of NF-*κ*B by upregulating endogenous regulatory molecules, including prohibitin, or by inducing more inhibitory subunits, I*κ*B, of NF-*κ*B, to keep them inactive in the cytoplasm (Figure 4).

The major event to exert anti-inflammatory effect is mediated via the nuclear translocation of corticosteroid-GR complex. The nuclear translocation signals responsible for nuclear transport and retention of activated GR are NL1 and NL2, where NL1 is similar in sequence to Simian virus 40 and the NL2 of GR is poorly defined [55, 56]. These nuclear localization signals are present on importins (Figure 4). Additionally, their nuclear export is mediated by chromosomal region maintenance (CRM-1) pathway.

Several importins have been found to be involved in the nuclear translocation of corticosteroid-GR complex. NL1 of GR binds to importin *α* and is responsible for importing corticosteroids into the nucleus. In addition, the other importins involved in nuclear translocation of corticosteroid-GR complex include importin 7, importin 8, and importin 13 [29, 48, 57, 58]. However, whether the NLS1 sequence, to which these importins bind, is the same or different is not very well understood [55] even though the presence of both NLS1 and NLS2 on GR and its binding to importin-7 has been found in nonmammalian cells, suggesting the crucial role of both NLSs for translocation of GR into nucleus [55]. Importin *β* stimulates the binding of GR to importin *α*, whether it is involved in the translocation of GR into the nucleus is poorly defined.

Recently, Hakim and colleagues [58] confirmed earlier findings of Xiao et al. [48] that importin 7 is critical in the translocation of glucocorticoid receptor from the cytoplasm into the nucleus (Figure 4). Also, the expression of the cofactor of importin 7 complex, RanGTP, is reduced during oxidative stress-induced corticosteroid insensitivity, as shown in the presence of hydrogen peroxide [58]. Interestingly, the degree of loss of importin 7 correlates well with the reduction in GR nuclear translocation and insensitivity of corticosteroids [58]. In several cases of severe asthma and chronic obstructive pulmonary disease (COPD), corticosteroids are not very effective [59]. In chronic inflammation, biomarkers of oxidative stress such as oxidant hydrogen peroxide and 8-isoprostane are upregulated, leading to exacerbated inflammation. This uncontrolled oxidative stress results in glucocorticoid insensitivity in the biosystem [60]. The diminished expression of importin 7 can knock down the protective effect of glucocorticoid receptors in human macrophages cell line, which might support the critical role of importin 7 in transporting GR from cytoplasm to the nucleus [58]. However, whether or not the insensitivity of corticosteroids in severe asthmatic and COPD patients is due to the defect in importin 7 is not known and warrants further studies in allergic subjects.

One of the mechanisms of corticosteroid-induced inhibition of Th2 response could be due to the competition between GR and GATA-3 to bind to importin *α* since both GR and GATA-3 utilize this karyopherin. Maneechotesuwan et al. [60] reported that fluticasone, a synthetic corticosteroid, either under* in vitro* conditions or upon inhalation causes suppression in nuclear transport of GATA-3 in human T lymphocytes. In this study, the interaction between GR and importin-*α* was found to be very effective even at low concentration of fluticasone. There was a reduction in GATA-3-importin *α* complex and an increase in GR-importin-*α* interactions when fluticasone was inhaled by asthmatic patients [60].

GR can also be carried into the nucleus by importin 7 and importin 13 [55, 58, 59] (Figure 4). Since NF-*κ*B and AP-1 activity can also be inhibited by GR, it is likely that GR has a higher affinity for the karyopherins than the inflammatory transcription factors and/or activated GR induces the production of endogenous inhibitors of importins involved in the translocation of inflammatory transcription factors. Therefore, any defect either in the activation of GR or its translocation into the nucleus could be responsible for the lack of responsiveness or decreased response to corticosteroids. On the other hand, there could be variants of importins that enhance the entry of GR to the nucleus and thus enhance the response to corticosteroids. Indeed, a genetic variant in importin 13 has been found to be associated with improved PC_20_ (i.e., decreased airway hyperresponsiveness) to methacholine in mild-to-moderate asthmatic children [61]. Accordingly, potential polymorphisms in various importins in allergic subjects could dictate proinflammatory or anti-inflammatory response.

## 9. Outstanding Questions and Future Directions

**Figure 5:**
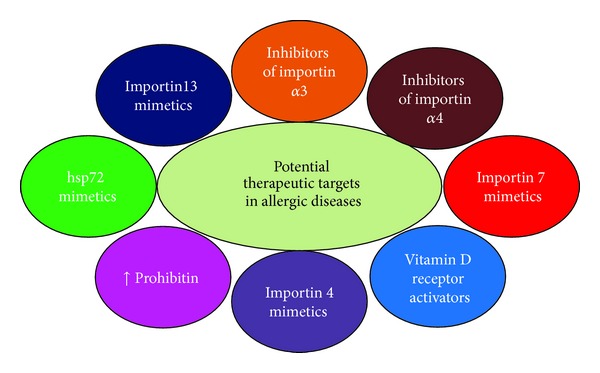
Schematic diagram showing various target sites for intervention in allergic diseases: there are many sites in the importin-exportin system in inflammatory cells involved in allergic diseases. Detailed knowledge on the involvement and role of specific importin molecule and/or the synthesis of mimetics/activators of endogenous inhibitors would help in the development of better therapeutic approaches in allergic diseases.

Since the nuclear import and export of the proteins occur in highly systematic and organized manner, it is critical to understand the precise regulatory mechanisms of the importin and exportin molecules with respect to their synthesis and function. This would assist in targeting specific sites to allow for the development of better therapeutic approaches in allergic diseases (Figure 5). Since several importin molecules are nonspecific in nature, antagonists or antibodies specifically against a particular importin might not prove to be clinically beneficial. However, additional knowledge regarding the precise control over the recruitment and activation of a few key karyopherins could be beneficial in the development of better therapeutic approaches. In addition, further studies regarding the absence and presence of endogenous mediators that regulate karyopherins in clinical allergic conditions would be informative. Potential polymorphisms and genetic variants in key importin molecules and the epigenetic control of karyopherins are also wide open fields for further investigation (Figure 5). At present, there are no studies on the nucleocytoplasmic shuttling of Foxp3 and T-bet, key transcription factors associated with allergic diseases. Overall, the potential role of importins and exportins in the regulation of allergic immune response is indeed both fascinating and challenging and thus warrants further investigation.

## Figures and Tables

**Table 1:** Names and symbols of human importins and exportins.

Transporters	Alternative name/s	Short name/s	NCBI symbols
Importin *α*1/ Importin *α*2	Karyopherin *α*2 RAG cohort protein 1 SRP1-*α*	RCH1 SRP1	KPNA2

Importin *α*3	Importin *α* Q1 Karyopherin *α*4	Qip1	KPNA4

Importin *α*4	Importin *α* Q2 Karyopherin *α*3	Qip2 SRP-1*γ*	KPNA3

Importin *α*5	Karyopherin *α*1 Nucleoprotein interactor 1 RAG cohort protein 2	NPI-1 SRP1-*β* RCH2	KPNA1

Importin *α*6	Karyopherin *α*5	—	KPNA5

Importin *α*7	Karyopherin *α*6	IPOA7	KPNA6

Importin *β*1	Importin 90 Karyopherin *β*1 Nuclear factor p97 Pore targeting complex 97 kDa subunit	PTAC97 NTF97	KPNB1

Importin *β*2	Transportin 1 Karyopherin *β*2 M9 region interaction protein	KPNB2 MIP1 TRN	TNPO1

Importin *β*3	Importin 5 Karyopherin *β*3 Ran-binding protein 5	Imp5 KPNB3 RanBP5	IPO5

Importin 4	Importin 4b Ran-binding protein 4	Imp4b RanBP4 IMP4B	IPO4

Importin 7	Ran-binding protein 7	Imp7 RanBP7	IPO7

Importin 8	Ran-binding protein 8	Imp8 RanBP8	IPO8

Importin 9	Ran-binding protein 9	Imp9 RanBP9 KIAA1192 HSPC273	IPO9

Importin 11	Ran-binding protein 11	Imp11 RanBP11	IPO11

Importin 12	Transportin 3 Transportin-SR	Imp12 TRN-SR IPO12	TNPO3

Importin 13	Karyopherin 13	Kap13 RanBP13 KIAA0724	IPO13

Transportin 2	Karyopherin *β*2b	—	TNPO2

Exportin 1	Chromosome region maintenance 1 protein homolog	Exp1 CRM1	XPO1

Exportin 4	—	Exp4 KIAA1721	XPO4

Exportin 5	Ran-binding protein 21	Exp5 KIAA1291 RanBP21	XPO5

Exportin 6	Ran-binding protein 20	Exp6 KIAA0370 RanBP20	XPO6

Exportin 7	Ran-binding protein 16	Exp7 KIAA0745 RanBP16	XPO7

Exportin t	tRNA exportin	—	XPOT

**Table 2:** Specificity of different classes of importins/exportins in transporting cargo to and from the nucleus.

Transporters	Organism	Cells/Tissue	Cargo (proteins) specific to each importin or exportin	Reference
Importin *α*1	Human	ROS 17/2.8, UMR-106, MC3T3-E1, and SaOS-2	Type 1 parathyroid hormone receptor	[62]

Importin *α*2	Human	HuT-78 cells	GATA-3	[63]

Importin *α*3	Human	A5A9 lung cells, human Bronchial smooth muscle cells	NF-*κ*B p50/p65	[25, 50]

Importin *α*3	Mouse	Mouse lung tissue	NF-*κ*B p65	[11]

Importin *α*4	Human	A5A9 lung cells	NF-*κ*B p50/p65	[25]

Importin *α*5	Human	COS-1 HepG2 HuH7 hepatoma cells	STAT3 STAT1-STAT2	[45] [43]

Importin *β*1	Human	HEK293 HUVEC C58 CO57 HeLa	PRPF31 NF-*κ*B CREB PTHrP SRY/SOX-9 Cyclin B1 NFAT	[64–69] [33] [68]

Importin *β*1	Mouse	Sf9	NFAT	[69]

Importin *β*2	Human	HeLa	HPV16E6 oncoprotein	[70]

Importin *β*3	Human	HeLa	c-Jun Ribosomal proteins	[71, 72]

Importin 4	Human	HeLa	Vitamin D receptor HIF1-*α*	[73, 74]

Importin 5	Human	HeLa	c-Jun	[70]

Importin 7	Human	HeLa	c-Jun Histone H1 CREB SMAD3 Glucocorticoid receptor	[71] [75, 76] [33] [48] [55]

Importin 8	Human	HaCaT COS-1	SMADs Signal recognition particle protein 19	[76, 77]

Importin 9	Human	HeLa	c-Jun PP2A (PR65)	[71] [78]

Importin 11	Human	BHK	L12 UbcM2	[79, 80]

Importin 13	Human	HeLa Airway epithelial cells	c-Jun Glucocorticoid receptor	[71] [57]

Importin *α*/*β*	Human	HTC MCF-7	HIV-1 integrase P53	[81, 82]

Exportin 1	Human	NIH-3H3 HeLa	Cyclin D1 I*κ*B NFAT	[14, 26, 83, 84]

Exportin 4	Mouse	HeLa	elF-5A	[15]

Exportin 5	Human	BHK	Staufen2	[85]

Exportin 6	Human		Actin	[86]

Exportin 7	Human	HeLa BHK	P50RhoGAP	[87]

Exportin t	Human	HeLa	tRNA	[72, 88]
